# Does Antigen Glycosylation Impact the HIV-Specific T Cell Immunity?

**DOI:** 10.3389/fimmu.2020.573928

**Published:** 2021-01-22

**Authors:** Alex Olvera, Samandhy Cedeño, Anuska Llano, Beatriz Mothe, Jorge Sanchez, Gemma Arsequell, Christian Brander

**Affiliations:** ^1^ IrsiCaixa—AIDS Research Institute, Badalona, Spain; ^2^ Universitat de Vic–Universitat Central de Catalunya (UVic-UCC), Vic, Spain; ^3^ Fundació Lluita contra la Sida, Infectious Diseases Department, Hospital Universitari Germans Trias i Pujol, Badalona, Spain; ^4^ Centro de Investigaciones Tecnológicas, Biomédicas y Medioambientales, Universidad Nacional Mayor de San Marcos, Lima, Perú; ^5^ Institut de Química Avançada de Catalunya (IQAC-CSIC), Barcelona, Spain; ^6^ Institució Catalana de Recerca i Estudis Avançats (ICREA), Barcelona, Spain

**Keywords:** virus, human immunodeficiency virus, T cell, cytotoxic T lymphocyte, epitope, glycosylation

## Abstract

It is largely unknown how post-translational protein modifications, including glycosylation, impacts recognition of self and non-self T cell epitopes presented by HLA molecules. Data in the literature indicate that *O*- and *N*-linked glycosylation can survive epitope processing and influence antigen presentation and T cell recognition. In this perspective, we hypothesize that glycosylation of viral proteins and processed epitopes contribute to the T cell response to HIV. Although there is some evidence for T cell responses to glycosylated epitopes (glyco-epitopes) during viral infections in the literature, this aspect has been largely neglected for HIV. To explore the role of glyco-epitope specific T cell responses in HIV infection we conducted *in silico* and *ex vivo* immune studies in individuals with chronic HIV infection. We found that *in silico* viral protein segments with potentially glycosylable epitopes were less frequently targeted by T cells. *Ex vivo* synthetically added glycosylation moieties generally masked T cell recognition of HIV derived peptides. Nonetheless, in some cases, addition of simple glycosylation moieties produced neo-epitopes that were recognized by T cells from HIV infected individuals. Herein, we discuss the potential importance of these observations and compare limitations of the employed technology with new methodologies that may have the potential to provide a more accurate assessment of glyco-epitope specific T cell immunity. Overall, this perspective is aimed to support future research on T cells recognizing glycosylated epitopes in order to expand our understanding on how glycosylation of viral proteins could alter host T cell immunity against viral infections.

## Introduction

Since the early years of the human immunodeficiency virus (HIV) pandemic, it has been noticed that not all HIV-1 infected individuals show equally fast disease progression to acquired immunodeficiency syndrome (AIDS) (1). It is now well-recognized that a small proportion of HIV infected people can maintain low or even undetectable levels of plasma viremia for a long time in the absence of antiretroviral treatment. This population of long term non-progressors has been extensively studied, with the intention to identify immune correlates of controlled HIV replication and to develop an effective HIV vaccine. The immunological mechanisms that allow superior HIV infection control are not fully understood (2–4), but some HIV-specific CD8+ cytotoxic T lymphocyte (CTL) responses have been consistently associated with HIV viral set point. In line with this, different human leukocyte antigen (HLA) alleles have been related to HIV virus control or disease progression (4–6) and major efforts have been made to fine map HLA-restricted epitopes targeted by virus-specific CTL responses across the entire viral proteome (7). However, recent studies indicate that this impressive amount of information is possibly still lacking a significant portion of the full HIV epitope landscape (8, 9). One potential gap in the current knowledge of the CTL response to HIV is the potential existence of HLA class I restricted epitopes containing post-translational modifications (PTM) derived from HIV proteins. There are many types of PTM, we will focus herein on the most abundant, glycosylation, and the existence of HIV-specific T cell responses to glycosylated epitopes (“glyco-epitopes”). T cell responses to such glyco-epitopes have been described in tumors (10–14), tuberculosis (15) and other viruses (16–18), but to our knowledge only in two studies for HIV (19–21).

## Does Glycosylation Have an Impact on Epitope Presentation?

Eukaryotic cell proteins can undergo two main types of protein glycosylation: i) *N*-glycosylation of asparagine residues and ii) *O*-glycosylation of serine and threonine residues, which can be α- or β-*O*-linked (22). Glycosylation enzymes are thought to be highly compartmentalized which explains why the cellular localization of a protein can determine its glycosylation profile. *N*- and α-*O*-glycosylation are thought to occur predominantly on secreted proteins, whereas β-*O*-glycosylation affects nuclear and cytosolic proteins (23, 24). Of importance for T cell reactivity to glyco-epitopes, several studies have documented that glycans can survive the antigen processing and presenting process. In the late 1990s, CD4+ and CD8+ T cells that specifically recognized peptides carrying mono- or disaccharides where isolated (19, 25–32). The existence of HLA class I presented epitopes was further supported when it was shown that 0.1% of all peptides bound to HLA class I carried *O*-linked GlcNAc residues (33, 34). However, this number could be an underestimation since the epitope elution process can cause the stripped glyco-epitopes to lose their sugar moieties (35).

Despite this evidence of HLA-presented glyco-epitopes, the *in vitro* demonstration of reactive T cell responses has been limited, since most research on T cell responses and epitope mapping has employed only synthetic peptides to stimulate T cells. Such synthetic peptides do not carry any PTM and T cells targeting glyco-epitopes will thus not be detected. Alternatively, epitope mapping studies have used recombinant proteins or viral vectors expressing the antigen of interest (e.g. adenovirus or vaccinia virus). In recombinant proteins, glycosylation could be present if eukaryotic systems were used to produce them, while antigens expressed off viral vectors could be glycosylated by the host cells. In both cases the sugar residues added could possibly match the ones seen in the native protein, but this may only be the case if the same intracellular protein trafficking pathways are targeted (36).

These considerations indicate that in theory, protein glycosylation could affect antigen-specific T cell responses in several ways: i) epitope residues modified by glycosylation could be loaded onto HLA class I and recognized by the T cell receptor (TCR), but would be missed when using non-glycosylated peptide stimulations *in vitro*. ii) Glycosylation of epitope residues could mask proteolytic sites from proteasome digestion, HLA anchor residues or TCR binding residues, offering viruses an escape strategy to avoid CTL immune recognition.

Indeed, crystal structure analyses of MHC/glyco-peptide/TCR complexes indicate that MHC binding is mediated by the peptide backbone, while the glycan moieties interact with the TCR variable sites (17, 25, 37, 38). In a report by Avci et al. (39, 40), a carbohydrate CD4+ T cell epitope derived from a streptococcal glyco-conjugate was found to significantly increase vaccine-induced T cell responses. Also, the presence of a sugar moiety was tolerated by T cells, except when the glycosylation affected the epitope anchor residues (27–30). Still, Apostolopoulos et al. showed that in some cases, the MHC class I binding pocket itself could also accommodate an α-*O*-linked GalNAc (41). This glyco-epitope elicited CTL responses and was capable of cross-reacting with the non-glycosylated counterpart as its structure could be superimposed with a peptide showing a canonical anchor (42). Indeed, some data indicate that the smaller *O*-glycans may be more readily tolerated by T cell receptors than the larger *N*-glycans and that the central CDR3 region of αβTCR cannot accommodate more than four sugars (43). Whether this is rather the exception than the rule and which HLA complexes could accommodate glycosylated anchor residues on presented epitope remains to be clarified (31, 44). It is however interesting to note that several HLA class I alleles use anchor residues that could potentially be glycosylated, including: A*01, A*26, A*30:04, A*34:02; A*66, A*68, A*69, B*15:16, B*15:17, B*40, B*57:01, B*57:02, B*58:01, and B*58:02, (http://www.syfpeithi.de/bin/MHCServer.dll/FindYourMotif.htm). Six of these alleles (B*15’s, B*57’s, and B*58’s) have been associated with superior control of HIV infection *in vivo* (4). Yet, no study has addressed how and whether glycosylation at anchor residues could affect epitope binding and recognition by HIV-specific T cells.

To date, most studies on HIV protein glycosylation have been focused on the envelope protein (Env) and its relationship with viral escape from humoral immunity (45). As other viruses, HIV is highly dependent on the host cellular machinery and extensive glycosylation of viral proteins has been documented (18, 23, 46, 47). In addition, there are several reports that suggest that HIV could interfere with the host glycosylation machinery (45, 48–54), but only two (19, 21) describe T cell responses to glycosylated epitopes.

Together, the available studies indicate that glycosylated epitopes can be presented by HLA molecules and that T cell responses directed against glyco-epitopes can be induced *in vivo* (35, 55, 56). It is tempting to speculate that, if HIV glyco-epitope specific T cell responses exist and are restricted by HLA class I alleles that are associated with superior HIV control *in vivo*, they could contribute to HIV control. Strikingly, they would have been largely missed by the use of synthetic non-glycosylated peptides to screen for T cell responses.

## Are HIV Peptides Containing Predicted Glycosylation Sites Less Frequently Targeted by T Cells?

**Figure 1:**
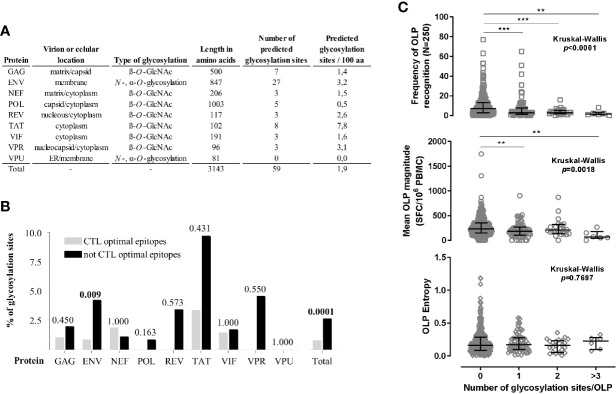
Potential impact of glycosylation on T cell responses to HIV. **(A)** Predicted glycosylated positions in HIV proteins taking into account cellular location of viral proteins. **(B)** Percentage of predicted glycosylated sites in known, optimally defined CTL epitopes (9) in comparison to regions for which no optimal CTL epitopes have been defined. Frequency differences were analyzed by the Fisher exact test, p<0.05 are highlighted in bold numbers. **(C)** Frequency of recognition and magnitude of the response to HIV OLP in 250 HIV infected individuals stratified by the number of potential glycosylations in each OLP. The existence of statistically significant differences was analyzed using the Kruskal-Wallis test, Mann-Whitney *p* values are indicated by *p<0.05, **p<0.01, ***p<0.001, ****p<0.0001.

To establish evidence for potential effects of glycosylation on T cell recognition of HIV derived epitopes, we assessed whether HIV protein fragments containing predicted glycosylable positions were less frequently targeted by T cells than the rest of the viral proteome. *N-*linked oligosaccharides are covalently attached to glycoproteins on asparagine residues within the Asn-X-Ser/Thr sequence motif (where X is any amino acid residue except proline) (57) and can be fairly well predicted *in silico*. In contrast, O-glycans have no single consensus sequence, although most frequently occur on serine or threonine residues. Current prediction tools use artificial neural networks that examine the sequence context of glycosylable amino acids to predict them (23, 58, 59). We used NetNGlyc (*N*-glycosylation sites), NetOGlyc (mucin type GalNAc *O*-glycosylation) and YinOYang (*O*-β-GlcNAc attachment sites) in the CBS website (http://www.cbs.dtu.dk/services/), to predict 87 glycosylation sites in the HIV clade B 2001 consensus protein sequences (https://www.hiv.lanl.gov/content/sequence/NEWALIGN/align.html). Since the type of glycosylation that a protein can undergo is highly depended on cellular location, we used protein location to reduce these predictions to a total of 59 glycosylation sites (**Figure 1A**). The distribution of these 59 sites could affect T cell epitopes distributed over a range of approximately 1000 amino acids of the virus and affect thus T cell immunity to a third of the viral proteome. Overall, Gag and Tat proteins showed the highest number of glycosylable positions, with Tat containing the highest density (8 glycosylable positions/100 amino acid, **Figure 1A**).

To investigate whether these sites could indeed be involved in the T cell response to HIV infection, we reanalyzed existing T cell response data (2, 60), adding the predicted glycosylation sites across all HIV proteins into the analysis. We asked whether the potential glycosylation sites fell within previously described CTL epitopes or in regions in which screenings using non-glycosylated synthetic peptides have shown little T cell reactivity. For this, we used the Optimal HIV CTL epitope list at the Los Alamos HIV Immunology Database (http://www.hiv.lanl.gov/) curated by our laboratory to define epitope-rich regions (9). We found a statistically significant underrepresentation of glycosylation sites among all the optimally defined CTL epitopes compared to the rest of the viral proteome (Fisher exact test *p*=0.0001, **Figure 1B**). This trend still held true for all viral proteins analyzed individually (except Nef), with statistical significance maintained for the Env protein (*p*=0.009).

In a second step we used the frequency of recognition and the magnitude of response to 410 overlapping peptides (OLP), spanning the entire viral proteome, in a cohort of 250 clade B HIV chronically infected subjects previously tested in our laboratory (2, 61). The OLP reactivity data were stratified by the presence of one or more predicted glycosylation positions within each OLP and compared to the frequency of OLP recognition. This analysis showed a strong inverse relationship between the presence of glycosylation site(s) in a given OLP and the frequency at which the OLP was targeted (**Figure 1C**, Kruskal-Wallis *p*<0.0001). In addition, when OLP containing glycosylation sites were targeted, they elicited responses of reduced magnitude (**Figure 1C**, Kruskal-Wallis *p*= 0.018). Together, these results indicate that fragments of the viral proteome containing potentially glycosylated positions are less frequently targeted by T cells - in studies using non-glycosylated synthetic peptides as stimuli. These observations suggest two different scenarios: (i) glycosylation sites are inherently poorly immunogenic because of the PTM or (ii) epitopes can contain glycosylated residues and are able to induce a T cell responses, but they have not been detected because essentially all T cell epitope screenings have been performed using non-glycosylated synthetic peptides. There are several arguments that give strong support to the second scenario: OLP with potential glycosylation sites do not differ in entropy from the ones not containing these sites (**Figure 1C**). This indicates that the frequency of response to these regions was not underestimated because of increased sequence divergence between autologous virus and the OLP sequences used as recall antigen (62). In addition, there are studies that have shown different results when T cell responses to HIV were assessed using synthetic peptides or viral antigens expressed by a vaccinia virus, further supporting the hypothesis that some responses are detected only when the antigen is produced in a eukaryotic cell system, which allows PTM to occur, but not when using PTM-free synthetic peptides (63).

## Technical Limitations of Synthetic Glycopeptide Screens

Based on the previously referenced literature and the above *in silico* data, an INFγ ELISPOT or intracellular staining (ICS) screen, using synthetically glycosylated OLP as stimulus, would be the first-choice option to screen for HIV derived glyco-epitopes. Such an approach has been successful in detecting T cell responses to regular peptides but, in our hands poses a number of technical limitations when attempting to detect responses to glycosylated peptides. In particular, α-*O*- and *N*-glycosylation of proteins includes complex, highly branched, sugars that are challenging to approach by chemically synthesis of the corresponding glycopeptides. Moreover, glycoproteins suffer extensive de-glycosylation in the cytosol before entering the proteasome, making difficult to predict which α-*O*- or *N*-linked glycans will be present in the peptides eventually presented by an HLA class I molecule. This severely limits glycosylated peptide design, discouraging the use of α-*O*- or *N*-glycosylated peptides in T cell screens. However, β-*O*-GlcNAc glycosylation, involving the addition of a single *N*-acetylglucosamine (GlcNAc) to a Ser or Thr residue, is comparably much easier to approach experimentally. For this reason, we attempted to evaluate the effect of glycosylation on T cell responses using β-*O*-glycosylated synthetic peptides. Additionally, since N-glycosylation followed by complete *N*-de-glycosylation causes a change of an Asn (N) residue to Asp (D), we also tested OLP sequence variants that contained N to D substitutions to account for potential responses to *N-de-*glycosylated neo-epitopes (64).

**Figure 2:**
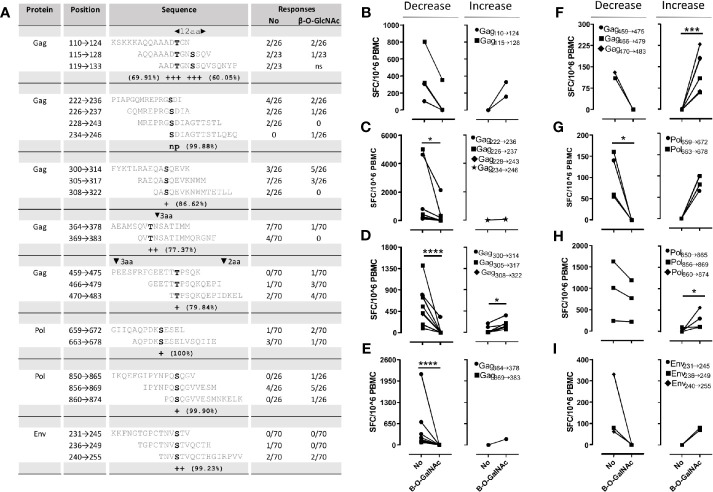
INFγ-ELISPOT screening with β-*O*-GlcNAc OLP and its non-glycosylated counterparts. **(A)** Frequency of responders to OLP with and without glycosylation modification. Presence of insertions or deletions is indicated in upper grey bars. Prediction of O-*β*-glycosylated positions by YingOYang is based on the server output (http://www.cbs.dtu.dk/services/). The frequency of the potential glycosylation sites in the HIV sequence database alignments is also indicated. **(B–I)** Magnitude of the response to *β*-*O*-glycosylated peptides and their non-glycosylated equivalents. OLP covering the same region are shown in the same graph, individuals who decreased their OLP response to the glycosylated OLP version are shown in the left panels while individuals with increased responses to the glycosylation containing OLP are shown in the right hand panels. Mann-Whitney *p* values are indicated by *p<0.05, **p<0.01, ***p<0.001, ****p<0.0001.

To perform this proof-of-concept analysis, OLP covering potentially glycosylated regions were designed using the 2010 compendium alignment of Gag, Pol, Env, and Nef from the Los Alamos HIV Sequence Database (https://www.hiv.lanl.gov/content/sequence/NEWALIGN/align.html). Seven regions in Gag, Env and Pol containing predicted β-*O*-glycosylation sites were selected for glycopeptide synthesis. Consensus sequence based OLP and the corresponding O-GlcNAc glycopeptides covering these regions (**Figure 2**) were synthesized using stepwise solid-phase peptide synthesis following standard Fmoc protocols using glycosylated serine and threonine building blocks (see Supporting information). Additionally, we synthesized OLP covering three potential *N*-glycosylation sites in Env and Gag with the original N residue or the D substitution, which can be caused by de-glycosylation of *N*-glycosylated positions during antigen processing. Two shorter (9mer), already described, epitopes containing *N*-glycosylation sites in Env, together with their D modifications were also produced (19). Four out of the five potentially *N*-glycosylated positions in these peptides (positions 88, 156, 160, and 301 in Uniprot entry P04578) have been demonstrated to be *N-*glycosylated experimentally (65–67). These peptides were used to screen HIV infected individuals for INFγ-producing T cell responses in peripheral blood mononuclear cells (PBMC) to potentially β-*O*-glycosylated or de-*N*-glycosylated epitopes using an INFγ-ELISPOT (Mabtech). We used samples from a total of 71 individuals (supporting information) including individuals with different HIV-infection status and representing 55 different HLA-A, -B, and -C alleles.

Globally, the frequency of responses to these peptides was low and precluded drawing of strong conclusions from the results. This low frequency of responses could be because glycosylated sequences are inherently poorly immunogenic, but also by a poor representation in the samples used of the HLA alleles capable of presenting glycosylated epitopes. Since these HLA alleles have not been identified and taking into account the vast number of different HLA alleles described, they could have been largely missed in this study. Overall, responses were more frequent (range: 0%–26%, **Figure 2A**) and of higher magnitude (range: 0–2715 SFC/10^6^ PBMC, Mann-Whitney *p*=0.0054) when targeting non-glycosylated peptides compared to reactions to their O-*β*-glycosylated counterparts (0%–19% and 0-560 SFC/10^6^ PBMC, respectively). Despite its low frequency, these results provide evidence that O-*β*-glycosylation can interfere negatively with epitope recognition. Indeed, analyzing each potentially glycosylable region individually, the magnitude of the response to O-*β*-GlcNAc modified peptides was usually lower or zero (*p*<0.05 in **Figures 2C–G**). However, responses to two regions in Gag and one in Pol showed a significant increase after glycosylation in some individuals (p<0.05 in **Figures 2D, F, H**). This suggested that, although they are rare, responses to O-*β*-glycosylated epitopes can be detected. We also screened 22 HIV infected individuals with the OLP containing potentially *N*-glycosylable positions (N) and its deaminated counterparts (D). We found only four responders and were unable to detect differences among the different versions of the peptides (data not shown).

## Future Perspectives

Our preliminary data, indicate that O-*β*-GlcNAc glycosylation usually reduces peptide recognition and suggest that sugar moieties in synthetic glyco-peptides often interfere with peptide binding to HLA or with TCR recognition. In the context of HIV infection, this would be in line with a mechanism where HIV could use glycosylation to escape from the T cell response, similarly to what happens with antibody recognition of the viral Env protein. Still, it seems that specific T cell responses to O-*β*-glycosylated peptides could exist, although they would be relatively rare and weak. It remains to be addressed what physiological role these responses may have in *in vivo* HIV control. As shown recently, inhibition of glycosylation in HIV producing cells leads to massive increase in virus replication; suggesting that the glycosylation of viral proteins comes at some fitness costs while possibly protecting from immune surveillance (46, 68). It will be interesting to assess the balance between such reduced replication fitness and the ability to use glycosylation as an escape strategy to avoid T cell immunity (if at all occurring *in vivo*) in future research. However, future studies will need more refined methodologies to identify glycosylated peptides that can be accommodated in the HLA-class I groove, the specific HLA alleles that can bind them and measure glyco-epitope specific T cell responses. The use of HLA peptide elution methodologies in combination with lectin columns will allow to specifically capture glyco-peptides, while identifying the specific HLA alleles that can present them. The exact molecular nature of the captured glyco-peptides can be then characterized by mass-spectrometry (13, 69–74). Independent synthesis of these newly identified HLA-binding glycopeptides, containing the specific *N-* or *O-*linked glycans characterized by mass-spectrometry, should allow identifying specific T cell responses in individuals bearing the HLA class I allele from where the glycosylated peptides were eluted from. Additional structural analyses will be needed as well, to better define the molecular structure of glyco-epitope glycan moieties, to identify carbohydrates that can block recognition by specific TCRs, but also to identify TCR that can accommodate such (complex) sugars. This improved focus should permit to accurately characterize glyco-epitope specific T cell responses and to identify interference with CTL recognition due to epitope glycosylation and relate both with HIV control. Filling this gap of knowledge might contribute to the development of CTL based vaccines, which may all depend on the proper glycosylation profiles of vaccine-delivered antigens. This may require adequate cellular expression and trafficking and may be especially important for the induction of vaccine-encoded antigens. However, our emerging data and the presented consideration also highlight an urgent need to better understand the impact of glycosylation on natural and vaccine-induced antiviral T cell responses.

## Data Availability Statement

The raw data supporting the conclusions of this article will be made available by the authors, without undue reservation.

## Ethics Statement

The studies involving human participants were reviewed and approved by Ethics Committee of the Hospital Universitari Germans Trias i Pujol, Badalona, Spain (PI-13-017). All participants provided written informed consent in accordance with the Declaration of Helsinki.

## Author Contributions

AO and CB performed data analysis and first manuscript writing. GA assessed *O*- and *N*-glycosylation metabolic routes and synthesized the *O*-GlcNAc peptides. AO, AL, and SC designed and performed INFγ-ELISPOT assays. JS and BM coordinated HIV-infected subject cohort. All authors contributed to the article and approved the submitted version.

## Funding

The present study was supported by grant PI12/00529 (AO) from the Instituto de Salud Carlos III, co-financed by the Fondo Europeo de Desarrollo Regional (FEDER) “Una manera de hacer Europa” and funding from the European Union’s Horizon 2020 research and innovation program under grant European AIDS Vaccine Initiative 2020 (EAVI2020) #GA681137 (CB). CB is a senior ICREA research professor. The work was also partly supported by the HIVACAT program, the Fondation Dormeur, Vaduz, (Liechtenstein) and an unrestricted gift by Rafael Punter.

## Conflict of Interest

The authors declare that the research was conducted in the absence of any commercial or financial relationships that could be construed as a potential conflict of interest.
