# Supplementary material for: Does Antigen Glycosylation Impact the HIV-Specific T Cell Immunity?
Source: Front Immunol. 2021 Jan 22;11:573928. doi: 10.3389/fimmu.2020.573928 (PMC7862545; doi:10.3389/fimmu.2020.573928)
Supplement: Supplementary file 1 [file DataSheet_1.docx]

**SUPPORTING INFORMATION**

**Does antigen glycosylation impact the HIV-specific T cell immunity?**

Alex Olvera^1,2^, Samandhy Cedeño^1^, Anuska Llano^1^, Beatriz Mothe^1,2,3^, Jorge Sanchez^4^, Gemma Arsequell^5^, Christian Brander^1,2,6^

^1^IrsiCaixa – AIDS Research Institute, Badalona, Barcelona, Spain

^2^Universitat de Vic–Universitat Central de Catalunya (UVic-UCC), Vic, Barcelona, Spain

^3^Fundació Lluita contra la Sida, Infectious Diseases Department, Hospital Universitari Germans Trias i Pujol, Badalona, Spain.

^4^Centro de Investigaciones Tecnológicas, Biomédicas y Medioambientales, Universidad Nacional Mayor de San Marcos, Lima, Perú.

^5^Institut de Química Avançada de Catalunya (IQAC-CSIC), Barcelona, Spain

^6^Institució Catalana de Recerca i Estudis Avançats (ICREA), Barcelona, Spain

**Material and methods**

**Peptide synthesis**

**Materials**

All amino acid building blocks, coupling reagents, and the corresponding prederivatized resins were purchased from Novabiochem AG. ACS grade organic solvents and other reagents were purchased from Sigma-Aldrich. All other reagents used were of analytical grade.

**Synthesis of glycosylated building blocks**

The glycosylated (GlcNAc) amino acids, FmocSer(O-β-D-Ac3GlcNAc)OH and FmocThr(O-β-D-Ac3GlcNAc)OH were prepared at the Institut de Química Avançada de Catalunya (IQAC-CSIC) following the methods reported by our laboratory (1). These compounds were derivatized as pentafluorophenol (pfp) esters following described procedures in the literature (2–4).


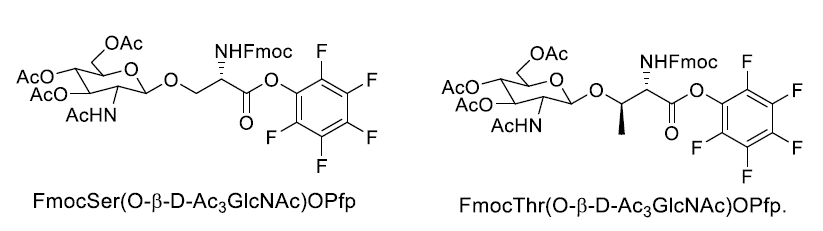


**Figure S1:** Chemical structured of the GlcNAc building blocks used in this work.

**Glycopeptide synthesis**

Glyco-peptides were prepared by stepwise solid-phase peptide synthesis following standard Fmoc protocols using glycosylated serine and threonine building blocks. Peptides and glycopeptides Gag_364-378_, _369-383_, _459-475_, _466-479_, _470-483_, Env_231-245_, _236-249_, _240-255_, Pol_659-672_ and _663-678_ were prepared at the Institut de Química Avançada de Catalunya (IQAC). Standard Fmoc solid-phase methodology on the corresponding Fmoc-amino acid prederivatized Wang resins were used. The amide couplings were performed by DIC (3 equiv) and HOBt (3 equiv) and Fmoc-protected amino acids (3 equiv) for 2 h at 25 ºC. The side chain protecting groups used to build the peptide sequences were the following: Trt for Gln and Asn, tBu for Ser and Thr, Boc for Lys, and Pmc for Arg. Coupling of Fmoc-O-(Ac3-β-D-GlcNAc)-L-Thr-OH (1.5 equiv) and Fmoc-O-(Ac3-β-D-GlcNAc)-L-Ser-OH (1.5 equiv) in DMF was carried out in the presence of DIC (1,5 equiv) and HOBt (3 equiv) for 2 h at 25 ºC. Washes between reactions were carried out with DMF and CH2Cl2. Completion of each coupling reaction was monitored by the Kaiser ninhydrin test. Intermediate capping steps were done (Ac2O, DIEA, CH2Cl2). Fmoc removal was achieved with piperidine–DMF (1:4) for 9 min. Washings between reactions were carried out with DMF and CH2Cl2. After chain assembly was completed, the glycopeptide resin was washed with DMF, CH2Cl2, and methanol. The resin was dried in a vacuum desiccator. O-Acetylated glycopeptides were cleaved from the resin with concurrent removal of the side-chain protecting groups by treatment with a cocktail of TFA–H2O-TIS (95:2.5:2.5) for 2 h at 25 ºC. The cleaved resin was then washed extensively with TFA (3 · 0.5 mL), the combined filtrates were evaporated. The crude peptides and O-acetylated glycopeptides were precipitated with ice-cold tert-butyl methyl ether, centrifuged, filtered, re-dissolved in water, and lyophilized. The final peptides were purified on a reversed-phase (C-18) column with the VersaFlashTM flash chromatography system using a H2O−ACN gradient and the correct fractions were pooled and lyophilized. The homogeneity and identity of the final peptides were determined by analytical RP-HPLC. All the obtained final peptides showed >98% purity. The purified peptides were characterized by analytical HPLC and by MALDI-ToF-MS. Analytical RP-HPLC were performed using the following solvents A (0.1% TFA in H2O) and B (0.1% TFA in acetonitrile) and the Nucleosil 100 RP-18 (5μm) C18 column (4x 250 mm). Analytical RP-HPLC programs used (gradients): TIPIC (From 90%A to 10%A in 30 min); GG2 (From 80%A to 20%A in 30 min). A final step was required to deprotect the protecting groups of the carbohydrate moiety of the O-acetylated glycopeptides (Zemplen deacetylation). The lyophilized material was then dissolved in methanol (5 mg/mL) for removal of O-acetyl groups as applicable. Sodium methoxide (0.14 M) in methanol (0.4–0.5 mL) was added dropwise to the glycopeptide solution. Depending on the sample, the pH was between 9 and 10 (as detected by pH paper), and the final concentration of NaOMe was between 10 and 15 mM. The O-deacetylation reaction was monitored by analytical RP-HPLC. Deprotection time varied among different glycopeptides. Neutralization was carried out by addition of acetic acid until pH 6 was reached. Pure glycopeptides were obtained after reversed-phase (C-18) column with the VersaFlashTM flash chromatography system using a H2O−ACN gradient and lyophilization. Glycopeptides Pol_856-869_, _850-865_, _860-874_, Gag _110-124_, _115-128_, _119-133_, _222-236_, _226-237_, _228-243_, _234-246_, _300-314_, _305-317_, _308-322_ were synthetized by a Custom Peptide Synthesis Services (Thermo Fisher Scientific GmbH, Ulm, Germany) using the building blocks produced at IQAC-CSIC as Pfp esters. The corresponding peptide sequences were also acquired from custom peptide synthesis services (ThermoFisher Scientific GmbH). The purified peptides were characterized by mass spectrometric analysis by the quality assurance service of Thermofisher Scientific.

**Peripheral Blood Mononuclear Cells (PBMC)**

PBMC were isolated from whole blood samples of HLA typed donors by using a Lymphoprep™ (Stemcell) density gradient and were cryopreserved until use. Samples from 68 chronic HIV infected donors and 3 seronegative individuals were used, representing 55 different HLA-A, -B and -C alleles. The sampled individuals represented different HIV infection status: 9 non-controllers (viral load: 3500-610000 HIV copies/ml, CD4: 25-834), 23 controllers (viral load:<25-1500 HIV copies/ml, CD4 counts 326-1525), 13 cART treated, 23 HIV-infected individuals for whom viral load and CD4 counts were unavailable, as well as 3 HIV seronegative donors. Before obtaining any samples, donors signed informed consent forms, following the Declaration of Helsinki. The study was carried out in accordance with the recommendations of the Ethics Committee of the Hospital Universitari Germans Trias i Pujol (Badalona, Spain).

**ELISPOT**

ELISPOT assay was performed by using a human IFN-γ ELISPOT kit (Mabtech AB) following the manufacturer’s instructions with minor modifications (5). For all assays, frozen PBMC were thawed and rested for 5 hrs at 37°C in RPMI1640 supplemented with a 10% of Fetal Bovine Serum (R10) medium before use. Cells were added at an input cell number of 1 × 10^5^ cells/well in R10 in 96-well polyvinylidene plates (Millipore Corp., Bedford, MA) coated with capture anti-INFg antibody DK-1. PBMC were stimulated with glycosylated peptides or their non-glycosylated counterparts (14 μg/ml final concentration for each peptide) for 16 hrs at 37°C in 5% CO2. Phytohemagglutinin (PHA) at 5 mg/ml (Sigma-Aldrich) was used as a positive control. R10 was used as negative control. The plates were developed using a biotin labeled anti-interferon antibody 7-B6-1, alkaline phosphatase (AP) labeled streptavidin and AP substrate kit (Bio-Rad Laboratories, Inc). The spots in each well were counted with an automated ELISPOT reader system (CTL Analyzers LLC) using ImmunoSpot software and the magnitude of responses was expressed as spot forming cells (SFC) per million PBMC. The threshold for positive responses was defined as at least 5 spots per well and responses exceeding the mean number of spots in negative control wells plus 3 standard deviations of the negative control wells and three times the mean of negative control wells, whichever was higher.

**Statistical analysis**

Statistical analysis was performed using GraphPad Prism version 10.0 for Windows. Non-parametric tests Mann–Whitney or Kruskal-Wallis tests were used to evaluate statistical significance of the differences among groups.

**References**

1. Arsequell G, Krippner L, Dwek RA, Wong SYC. Building blocks for solid-phase glycopeptide synthesis: 2-Acetamido-2-deoxy-β-D-glycosides of FmocSerOH and FmocThrOH. *J Chem Soc Chem Commun* (1994)2383–2384. doi:10.1039/C39940002383

2. Vargas-Berenguel A, Meldal M, Paulsen H, Bock K. Convenient synthesis of O-(2-acetamido-2-deoxy-β-D-glucopyranosyl)- serine and -threonine building blocks for solid-phase glycopeptide assembly. *J Chem Soc Perkin Trans 1* (1994)2615–2619. doi:10.1039/p19940002615

3. Beau J-M, Boyer F-D, Norsikian S, Urban D, Vauzeilles B, Xolin A. Glycosylation: The Direct Synthesis of 2-Acetamido-2-Deoxy-Sugar Glycosides. *European J Org Chem* (2018) **2018**:5795–5814. doi:10.1002/ejoc.201800735

4. Saha UK, Schmidt RR. Efficient synthesis of O-(2-acetamido-2-deoxy-β-D-glucopyranosyl)-serine and -threonine building blocks for glycopeptide formation. *J Chem Soc - Perkin Trans 1* (1997)1855–1860. doi:10.1039/a700210f

5. Mothe B, Hu X, Llano A, Rosati M, Olvera A, Kulkarni V, Valentin A, Alicea C, Pilkington GR, Sardesai NY, et al. A human immune data-informed vaccine concept elicits strong and broad T-cell specificities associated with HIV-1 control in mice and macaques. *J Transl Med* (2015) **13**: doi:10.1186/s12967-015-0392-5
